# A Highly Sensitive Enzyme-Amplified Immunosensor Based on a Nanoporous Niobium Oxide (Nb_2_O_5_) Electrode

**DOI:** 10.3390/s100505160

**Published:** 2010-05-25

**Authors:** Chang-Soo Lee, Dohyoung Kwon, Jeng Eun Yoo, Byung Gun Lee, Jinsub Choi, Bong Hyun Chung

**Affiliations:** 1 BioNanotechnology Research Center (BNRC), Korea Research Institute of Bioscience and Biotechnology (KRIBB), Daejeon 305-333, Korea; E-Mails: cslee@kribb.re.kr (C.-S.L.); kwondh@kribb.re.kr (D.-H.K.); 2 Department of Chemical Engineering, Inha University, 253 Yonghyun-Dong, Nam-Gu, Incheon 402-751, Korea; E-Mails: overstep2@nate.com (J.-E.Y.); byungdali@nate.com (B.-G.L.); jinsub@inha.ac.kr (J.C.)

**Keywords:** electrochemical biosensor, niobium oxide, enzyme, immunosensor

## Abstract

We report on the development of an enzyme-amplified sandwich-type immunosensor based on a thin gold film sputtered on an anodic nanoporous niobium oxide (Au@Nb_2_O_5_) electrode. The electrocatalytic activity of enzymatically amplified electroactive species and a stable electrode consisting of Au@Nb_2_O_5_ were used to obtain a powerful signal amplification of the electrochemical immunobiosensor. The method using this electrochemical biosensor based on an Au@Nb_2_O_5_ electrode provides a much better performance than those based on conventional bulk gold or niobium oxide electrodes. Our novel approach does not require any time-consuming cleaning steps to yield reproducible electrochemical signals. In addition, the strong adhesion of gold films on the niobium oxide electrodes offers a very stable substrate during electrochemical biosensing. Cyclic voltammetry measurements indicate that non-specific binding of proteins to the modified Au@Nb_2_O_5_ surface is sufficiently low to be ignored in the case of our novel system. Finally, we demonstrated the ability of the biosensor based on an Au@Nb_2_O_5_ offering the enhanced performance with a high resolution and sensitivity. Therefore, it is expected that the biosensor based on an Au@Nb_2_O_5_ has great potential for highly efficient biological devices.

## 1. Introduction

One of the most important factors in electrochemical immunosensing is the quality of the sensing electrode. During the development of highly sensitive and stable biosensors, one of the major goals is to create new types of electrodes that allow for fast and simple measurements of specific biological interactions. High-density surface functionalization, long-term stability of biomolecules, protection against non-specific binding, and proper biomolecular orientation to support simple and rapid specific interactions are strongly influenced by the types of electrode. Recently, Choi *et al.* reported that electrochemical signal enhancements that can easily distinguish between ssDNA and dsDNA can be realized by using a thin gold film sputtered on anodic nanoporous niobium oxide [1,2]. They claimed that their novel gold/metal oxide biosensor platform offers higher reliability and sensitivity compared to conventional electrodes.

In this paper, we report on a reliable, ultrasensitive enzyme-amplified electrochemical immunosensor that uses an enzyme to generate an electroactive product on a thin gold film sputtered on anodic nanoporous niobium oxide. Many types of electrochemical immunosensors have been developed during the past several decades for direct and specific measurement of very low protein concentrations. In particular, immunosensing techniques have been widely studied as a means to detect biochemically and biophysically specific interactions, such as antibody-antigen binding and protein-protein recognition [3–5]. Different types of labels including gold nanoparticles, liposomes and enzymes have been developed to amplify the electrochemical signal obtained from these specific interactions and to lower immunosensor detection limits [6–11].

One particularly attractive approach to electrochemical signal amplification combines enzymes with a further amplification step such as the redox cycling of enzymatically amplified electroactive species. In this technique, the electrochemical signal produced by biomolecular interaction is amplified by an enzyme that continuously generates electroactive products [9–11]. Such enzyme-amplified electrochemical immunosensors have been widely used for many miniaturized and microfluidic devices [12–15], which take advantage of the intrinsic simplicity, sensitivity, and robustness of electrochemical methods. In fact, the ease with which electrochemical instrumentation can be miniaturized makes the analytical approach eminently suitable for applications in portable bioanalytical micro- or nanodevices designed for simple, rapid, and high-throughput analyses of a small amount of target sample.

Here, we have measured the biospecific interaction between antigen and antibody with a sandwich type immunosensor using alkaline phosphatase (ALP) as the enzyme on the gold film coated nanoporous niobium oxide electrode. This allows us to determine the sensitivity of the electrochemical signal as a function of the mouse IgG concentration. Nanoporous niobium oxide offers good adhesion and a noticeable decrease in the detection limit for target molecules by amplifying the electrochemical signal via electrooxidation of *p*-aminophenol (AP) on the electrode. This is in part attributed to a structural stability of enzyme immobilized at the sputtered gold films and is in part to the higher roughness of the surface of the sputtered gold films. This method yields an electrochemical signal with twice the sensitivity of a bulk gold electrode. Along with the results reported by Choi *et al.* [1,2], this report demonstrates that Au@Nb_2_O_5_ can be a very useful electrode, not only for DNA sensors but also as protein sensors.

## 2. Experimental Section

### 2.1. Chemicals and Reagents

Mouse immunoglobulin G (IgG) from serum, biotinylated goat antimouse IgG, alkaline phosphatase (ALP)-congugated goat antimouse IgG, goat IgG form serum, 4-mercaptobenzoic acid (4-MBA), and 1-ethyl-3-(3-dimethylaminopropyl) carbodiimide hydrochloride (EDC) were obtained from Sigma. 4-Aminophenylphosphate (APP) was obtained from LKT Laboratories (Saint Paul, MN, USA). Bovine serum albumin (BSA) was purchased from Amresco Inc. (USA). The incubation buffer (IB) consisted of 50 mM Tris, 150 mM NaCl, and 1% BSA (pH 7.2). The rinsing buffer (RB) comprised 50 mM tris, 0.5 M NaCl, 0.05% Tween 20, and 0.05% BSA (pH 7.5). The buffer for electrochemical experiments (EB) consisted of 50 mM Tris, 10 mM KCl, and 1 g L^−1^ MgCl_2_ (pH 9.0).

### 2.2. Preparation of Anodic Nanoporous Niobium Oxide

The preparation of nanoporous niobium oxide by electrochemical anodization has been described elsewhere in detail [14–16]. In brief, Nb foil with a purity of 99.9% and a thickness of 0.25 mm (Goodfellow, UK) was cleaned by ultrasonication in acetone for 5 minutes, washed with ethanol and dried with a stream of N_2_ gas. Anodization was carried out at a constant voltage of 2.5 V in the mixture of 1 wt.% HF + 1 M H_3_PO_4_ at room temperature for 1 h, using a potentiostat/galvanostat (AutoLab PGSTAT12, Eco Chemie) interfaced to a computer. The cell was a three-electrode system consisting of a Pt mesh acting as the counter electrode, Ag/AgCl/3M KCl as the reference electrode and an Nb foil with a size of 1 cm^2^ as the working electrode. During the anodization, the stirring rate of the electrolyte was kept constant (∼180 rpm). As a result, nanoporous niobium oxide with average pore diameter of 10 nm, length of 120 nm and pore density of 4.6 × 10^15^/m^2^ was prepared. FE-SEM images of the anodic porous niobium oxide can be found in the reference [16]. Thin gold films were deposited onto nanoporous niobium oxide by a magnetron sputter (MSP-1S, Vacuum Device Inc., Japan), which is used for making an Au-coating layer and the thickness of gold layer was adjusted to 50 nm.

### 2.3. Surface Characterization

The prepared nanostructures were characterized by atomic force microscopy (AFM, Dimension 3,100, Veeco). The AFM measurement was carried out at the scan rate of 0.5 Hz on the size of 1 μm.

### 2.4. Preparation of an Immunosensing Layer

The Au@Nb_2_O_5_ was immersed in an ethanol solution of 1 mM 4-MBA for 12 h, washed with pure ethanol, and dried with N_2_ gas. The electrode was immersed in PBS buffer containing 50 mM EDC, 25 mM NHS and 100 μg mL^−1^ streptavidin for 2 h. The carboxylic groups were activated by EDC/NHS and sequentially attached streptavidin. After rinsing with RB, the streptavidin-modified electrode was incubated in IB for 30 minutes to prevent non-specific adsorption of proteins, and then washed with RB. The resulting assembly was immersed in IB containing 100 mg mL^−1^ biotinylated anti-mouse IgG for 40 minutes. After washing with RB, the target mouse IgG in IB was captured by the immunosensor for 40 minutes, followed by washing with RB. The immunosensor was finally incubated with 100 μL mL^−1^ ALP-conjugated anti-mouse IgG for 40 minutes and then washed with RB. The electrochemical experiment was performed using a potentiostat (ìStat 100, DropSens, Spain). The electrochemical cell consisted of the modified Au@Nb_2_O_5_ electrode, a Pt wire counter electrode and an Ag/AgCl reference electrode. The cell was filled with EB containing 1 mM APP. The APP solution was prepared daily. The electroactive area of the electrode is 0.271 cm^2^.

## 3. Results and Discussion

**Scheme 1.**
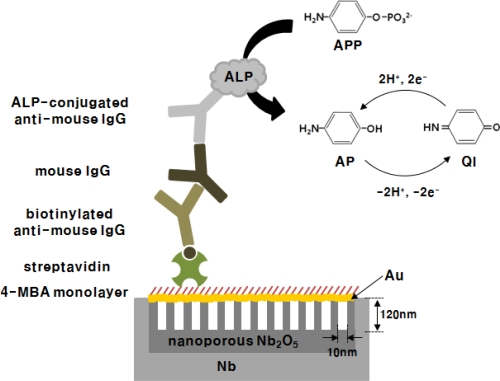
Schematic illustration of a sandwich type electrochemical immunosensor on thin gold films sputtered on nanoporous niobium oxide.

An electrochemical immunosensor for specific antibody–antigen interaction amplified by enzyme has been performed on a thin gold film sputtered on anodic nanoporous niobium oxide. Scheme 1 shows the immunosensing system which has been used in this work. The carboxylated 4-MBA self-assembled monolayer (SAM) was prepared on the Au@Nb_2_O_5_ electrode without any pretreatment, which provides an efficient site for immobilizing biomolecules to sensor surfaces. The highly compact SAM structure, which is formed by π stacking between molecules, obstructs direct electron transfer reaction and reduces the background current on the gold electrode [17,18].

**Scheme 2.**
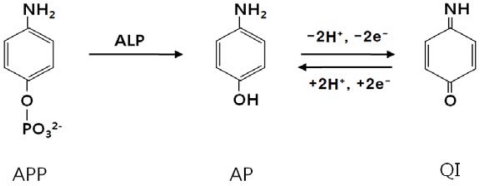
Electroactive generation of 4-aminophenylphosphate by alkaline phosphatase.

In this system, anti-mouse IgG-biotin antibody binds to streptavidin assay on the surface of 4-MBA monolayer and mouse IgG consequently. Then, the ALP-conjugated anti-mouse IgG antibody is specifically bound by antigen-antibody interaction. The utility of ALP as an electroactive label generator originates from an electrochemical enzyme immunosensor, in which an antibody or antigen-labeled enzyme acts on substrates such as APP, *p*-nitrophenyl phosphate, or phenyl phosphate and biocatalytically generates a redox-active product that can be detect using electrochemicalmethods (Scheme 2) [19–21]. Here, ALP enzymatically converts APP to electroactive aminophenol (AP).

To characterize the electrochemical signal generated from the immunosensing layer, anti-mouse IgG biotin antibody was bound to the streptavidin modified electrode, IgG mouse as a target followed by ALP-conjugated anti-mouse IgG antibody. Then, it was incubated for 1 minute in EB containing 1.0 mM APP.

### 3.1. Nonspecific Binding Conformation

In the presence of ALP, an oxidation peak usually appears at 0.2 V (*vs.* Ag/AgCl) due to redox mediated oxidation of AP. In contrast, such a peak is not observed in the absence of ALP-conjugated anti-mouse IgG antibody. This is explained by the following mechanism, where quinoimide (QI) is a molecule of AP that has been oxidized by loss of two electrons. Nonspecific protein adsorption is a well-known problem in heterogeneous sandwich immunoassays [22]. In order to investigate nonspecific adsorption of protein, 100 μL mL^−1^ ALP-conjugated anti-mouse IgG antibody was incubated for 40 minutes as following process: (a) Au@Nb_2_O_5_ electrode bound 4-MBA and the streptavidin, (b) Au@Nb_2_O_5_ electrode bound 4-MBA, the streptavidin and BSA, (c) Au@Nb_2_O_5_ electrode bound 4-MBA, the streptavidin, BSA and biotinylated anti-mouse IgG, (d) Au@Nb_2_O_5_ electrode bound 4-MBA, the streptavidin, BSA, biotinylated anti-mouse IgG and goat IgG, (e) Au@Nb_2_O_5_ electrode bound 4-MBA, the streptavidin, BSA, biotinylated anti-mouse IgG and mouse IgG, (f) the quantity of ALP-conjugated anti-mouse IgG antibody reduce 1/10 times than (e) electrode.

**Figure 1.**
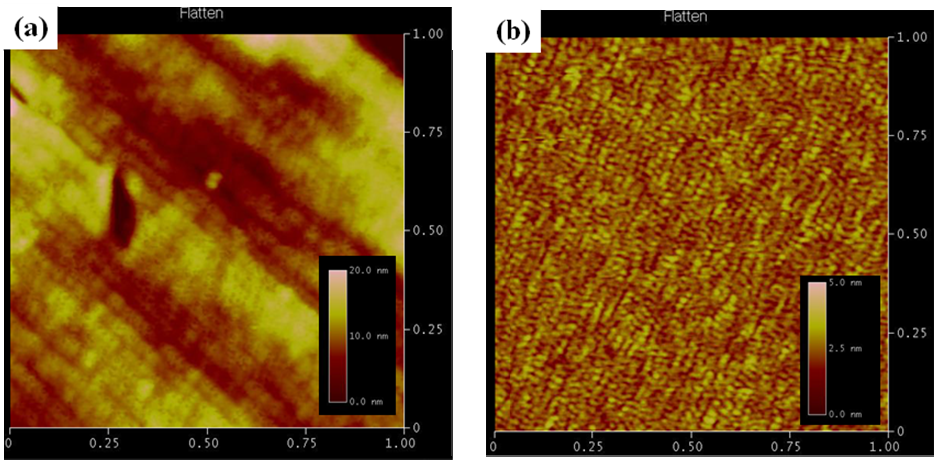
Atomic force microscope (AFM) topography of gold films sputtered on (a) nanoporous niobium oxide and (b) wafer. The sputtering of gold on both substrates was conducted for 30 s at 40 mA by a magnetron sputter (MSP-1S, Vacuum Device Inc., Japan).

Figure 1 shows the AFM topographies of gold films on nanoporous niobium oxide substrate and a flat silicon wafer, respectively. It clearly shows that the gold film on the nanoporous niobium oxide substrate has a higher surface roughness than that on the flat silicon wafer. As reported previously, the sputtered gold film offers reproducible results without the requirement of the time consuming cleaning steps. However, it was reported that the thin gold films sputtered on most substrates except nanoporous niobium oxide very easily peel off during immobilization or electrochemical measurement [2]. Thus, the nanoporous niobium provides not only higher surface roughness of the gold film but also stable adhesion of gold film.

**Figure 2.**
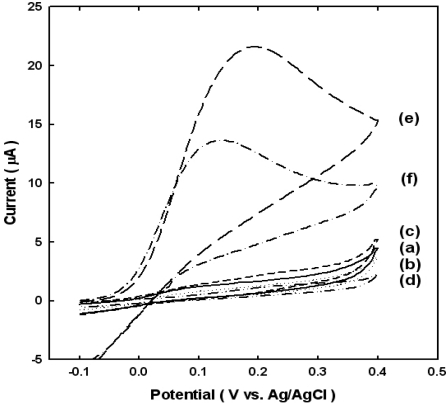
CV data of nonspecific binding conformation for various processes: (a) Au@Nb_2_O_5_ electrode bound 4-MBA and the streptavidin, (b) Au@Nb_2_O_5_ electrode bound 4-MBA, the streptavidin and BSA, (c) Au@Nb_2_O_5_ electrode bound 4-MBA, the streptavidin, BSA and biotinylated anti-mouse IgG, (d) Au@Nb_2_O_5_ electrode bound 4-MBA, the streptavidin, BSA, biotinylated anti-mouse IgG and goat IgG, (e) Au@Nb_2_O_5_ electrode bound 4-MBA, the streptavidin, BSA, biotinylated anti-mouse IgG and mouse IgG, (f) the quantity of ALP-conjugated anti-mouse IgG antibody reduce 1/10 times than (e) electrode. All CV were obtained after incubating for 1min in EB containing 1 mM APP and at a scan rate of 20 mV s^−1^.

Figure 2 shows cyclic voltammograms of the above biosensors, indirectly indicating that the emzymatic reaction amplifies the generation of electrochemically active molecules. The experimental results shown in Figure 2 demonstrate that the nonspecific adsorption, or namely the background current, is very small and negligible in our case. Since it is the extent of non-specific adsorption that usually determines the detection limit in these assays, we expect that the method described in this report can offer improvements of the detection limit.

As mentioned before, a peak at 0.2 V (*vs.* Ag/AgCl) does not appear in Figures 2(a–c) where the ALP-conjugated anti-mouse IgG antibody is not anchored because one of components for making the complete sandwich is omitted, whereas the oxidation peak is exhibited in Figures 2(e,f) where the sandwich is completed. Comparing to Figures 2(e,f), the electrochemical signal might increase in proportion to the amount of biocatalytically generated AP, because plenty of electroactive label (AP) are generated by the hydrolysis reaction of ALP. Thus we attempted to optimize the analyte signal by minimizing non-specific adsorption while maximizing sensitivity by ALP.

### 3.2. Calibration of Concentration Dependence

**Figure 3.**
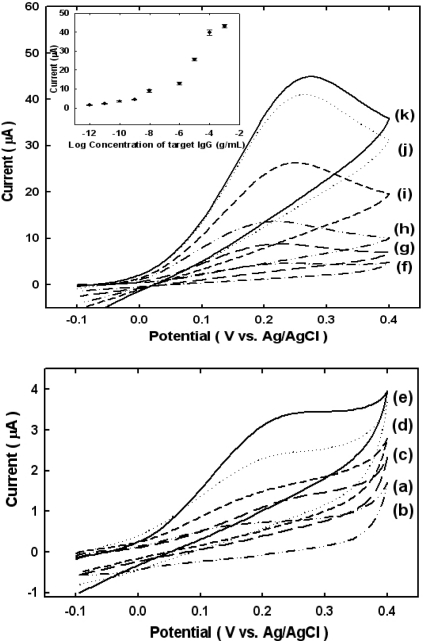
CV of a sandwich-type electrochemical immunosensor for mouse IgG at various concentrations of mouse IgG and goat IgG as negative control : (a) 100 μg mL^−1^ goat IgG, (b) 0 g mL^−1^, (c) 1 pg mL^−1^, (d)10 pg mL^−1^, (e) 100 pg mL^−1^, (f) 1 ng mL^−1^, (g) 100 ng mL^−1^, (h) 1 μg mL^−1^, (i) 10 μg mL^−1^, (j) 100 μg mL^−1^, and (k) 1 mg mL^−1^ mouse IgG. All CV were obtained after incubating for 1min in EB containing 1 mM APP and at a scan rate of 20 mV s^−1^ (In box, calibration plot obtained from the currents at 0.2 V. Currents are average for three electrodes.).

Figure 3 shows cyclic voltammograms of immunosensing electrodes obtained for various concentrations of mouse IgG in an EB solution containing 1 mM APP. We obtained at least three cyclic voltammograms at each concentration from 0 g mL^−1^ to 100 mg mL^−1^. The results show that increases in the peak current become visible when the concentration of mouse IgG is 1 pg mL^−1^ (see Figure 3(c)), and level off at concentrations of 1 mg mL^−1^ (see Figure 3 inset). This result indicates that the enzymatically enhanced electrochemical current strongly depends on the concentration of the target molecule, mouse IgG. As a negative control, we obtained a cyclic voltammogram when attempting to bind goat IgG rather than mouse IgG to biotinylated anti-mouse IgG (see Figure 3(a)).

The anodic current at 0.2 V in the cyclic voltammogram was plotted to determine the detection limit for mouse IgG. Generally, the detection limit is calculated by comparing the mean current at zero concentration with the mean current at three times the standard deviation. The calculated detection limit for mouse IgG was 1 pg mL^−1^, which may be useful in making real applications for biosensors.

### 3.3. Comparison with Bulk Gold Electrode

**Figure 4.**
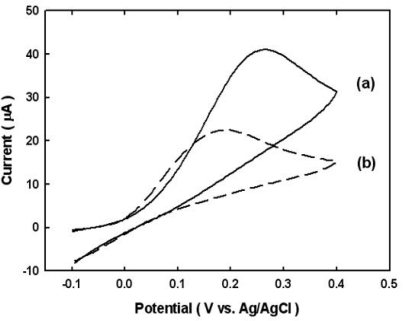
Comparison of CV data on (a) Au@Nb_2_O_5_ and (b) the bulk gold electrode. All CV were obtained after incubating for 1 minute in EB containing 1 mM APP and at a scan rate of 20 mV s^−1^. The concentration of mouse IgG is 100 μg mL^−1^.

Compared to the results obtained by using conventional bulk gold electrode immunosensors, this novel Au@Nb_2_O_5_ electrode improved the sensitivity of the measurement more than two-fold as, shown in Figure 4.

We speculate that the signal enhancement is due to enhanced activity associated with gold sputtered on the stable metal oxide. In addition, as demonstrated in the above experiments, the extent of non-specific biomolecular adsorption is very small. As a result, the electrochemical signal associated with the immunosensing reaction may be enhanced by an increase in specific adsorption. These findings may allow for the development of highly reliable and ultrasensitive electrochemical immunosensors that feature a very low detection limit for target molecules.

## 4. Conclusions

A highly sensitive enzyme-amplified electrochemical detection method using the specific biochemical recognition process between antibodies and antigens has been developed using a thin gold film sputtered on anodic nanoporous niobium oxide. We employed biotin/anti-biotin IgG as a model antigen/antibody pair. The use of our novel electrochemical immunosensor substantially improved critical performance parameters such as sensitivity and resistance to non-specific binding when compared to immunosensors based on more common bulk gold electrodes. Our results show that a thin gold film on anodic nanoporous niobium oxide can be used as a substrate for highly sensitive enzyme-amplified electrochemical immunosensors. We expect that the method can be adapted to offer sensitive and reproducible detection of a variety of clinically relevant proteins.
